# Assessment of root canal morphology of mandibular permanent anterior teeth in an Iraqi subpopulation by cone-beam computed tomography

**DOI:** 10.1016/j.jds.2021.02.010

**Published:** 2021-04-01

**Authors:** Ranjdar mahmood talabani

**Affiliations:** Conservative Department, College of Dentistry, University of Sulaimani, Madame Mitterand Street 30, Kurdistan Region, Sulaimani, 46001, Iraq

**Keywords:** Cone-beam computed tomography, Mandibular anterior teeth, Root canal morphology, Symmetry

## Abstract

**Background/purpose:**

Endodontic treatment is basically dependent on knowledge of the root canal anatomy. This study aimed to analyze the root canal configuration of mandibular anterior teeth in an Iraqi subpopulation using cone-beam computed tomography (CBCT) imaging.

**Materials and methods:**

A total of 305 CBCT scans involving 1794 mandibular permanent anterior teeth were evaluated. The number of roots, root canals, root canal system configuration according to Vertucci classification, and bilateral symmetry in root number and root canal number were recorded and statistically analyzed. The effects of gender and age on the incidence of root canal morphology and root canal number were investigated. Chi-square test was used to determine the level of significance (p < 0.05) and Kappa value was used to check reliability of results of the research.

**Results:**

Among the study patients, double root canals were found in (right 26.1% and left 26.4%) mandibular central incisors, in lateral incisors (right 28% and left 30.4%) and canines (right 11.7% and left 11%). Most teeth (77.8%) had a Type I Vertucci configuration and Type V was the least common and only present in canines (0.8%). All examined incisors presented with only one root and only around 2% of canines had two roots. Gender differences were identified for central incisors, while for lateral incisors and canines there were none. Patients older than 60 years had fewer teeth with double root canals (p < 0.05). Slight bilateral asymmetries appeared in central, lateral incisors, and canines in relation to root and root canal numbers.

**Conclusion:**

Single rooted with Type I canal configuration is the most prevalent in mandibular anterior teeth in the Iraqi subpopulation. However, the incidence of more than one root canal with different canal configurations is also detected.

## Introduction

Proper cleaning and filling of the root canal system and the consequent success of endodontic treatment is highly dependent on comprehensive understanding and knowledge of the complexity of the internal anatomy of teeth and its variation.^1^^,^^2^ Permanent mandibular anterior teeth typically present with a single root and a single canal. However, variety occurs in canal configurations, with additional canals or roots in the mandibular anterior teeth that may vary significantly with respect to ethnicity, race, and sex.^3^

Different methods were used in previous studies to examine the morphologic characteristics of the root canal system. Most of them were performed in vitro on extracted teeth using canal and modified canal staining, tooth clearing techniques,^4^^,^^5^ transverse cross-sectioning,^6^ scanning electron microscopy,^7^ and stereomicroscopy^8^ which are invasive techniques for identifying the configuration of canals. Radiography is one of the most important clinical tools in the detection of internal canal anatomy during endodontic therapy. However, conventional periapical radiography has limitations including distortion and superimposition of the root by adjacent structures due to its use of two-dimensional (2D) projections of 3D objects.^9^^,^^10^ With advances in technology, cone-beam computed tomography (CBCT) provides a 3D image of the teeth and surrounding structures in three orthogonal planes: axial, coronal, and sagittal. CBCT is considered more accurate than traditional radiography in the investigation of root canal configurations^6^ and as accurate as the modified canal staining and tooth clearing technique in identifying root canal systems.^11^ Also, it produces a significantly lower effective radiation dose compared with conventional computed tomography and is a noninvasive technique that provides high-quality images.^12^ Therefore, this study aimed to report the clinical incidence of the number of roots, root canals, and root canal configuration of mandibular anterior teeth in an Iraqi subpopulation using CBCT.

## Materials and methods

### The sample

A total of 305 patients, 122 (40.1%) males and 183 (59.9%) females, with mean age 40.1 years ranging from 18 to above 60 years of age, were included in this study. From these patients, 1794 mandibular permanent anterior teeth were evaluated (male: 720 teeth and female: 1074 teeth). The CBCT scans were retrieved from the database of the private B&R Dental Center, Sulaimani, Kurdistan Region/Iraq, for the period from January 2018 to June 2020. This study protocol was approved by the Ethics Committee of the University of Sulaimani College of Dentistry (No. 178). Only teeth with fully developed roots and closed apices were included in the study. The exclusion criteria for teeth were: previously treated root canals, apical periodontitis, calcification or internal or external resorption, caries or restoration, fiber post-restorations, and distorted CBCT images.

### CBCT examination

All CBCT images were acquired with a GALILEOS Sirona comfort PLUS unit (Sirona Dental Systems GmbH, Bensheim, Germany). Technical specifications were as follows: 15.4 cm spherical imaging volume, 0.25/0.125 mm isotropic voxel size, and a field of view of 15 cm diameter. The CBCT radiographs were taken according to the following parameters: 98 kVp, 3–5 mA, and exposure time of 14s by Sidex XG/Galileos implant software.

Each scan was assessed on-site using an identical step by-step screening protocol. A careful examination was obtained by optimal visualization using all software features, such as zooming, changes in contrast, and brightness.

The patients were divided into five groups (18–30 years, 31–40 years, 41–50 years, 51–60 years, and above 60 years of age). The gender (female or male) of the patients and the location of the included teeth were also recorded. The following parameters were evaluated:

1. The number of roots, the number of canals and root canal configurations on the basis of Vertucci's classification in the three planes (sagittal, axial, and coronal).
2. Differences between genders.
3. Bilateral symmetry of the number of roots and number of root canals.

**Figure 1:**
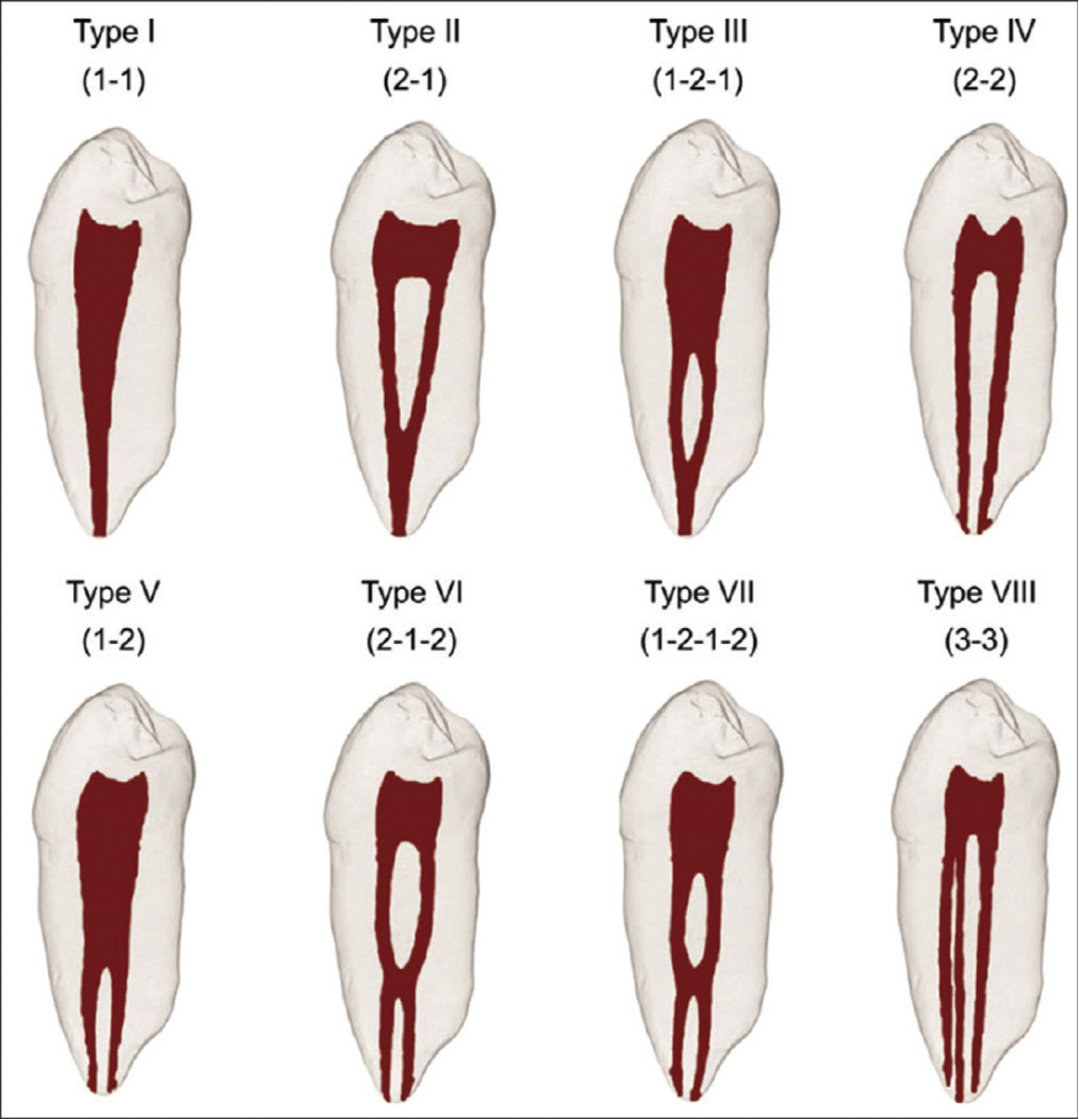
Diagrammatic representation of Vertucci's canal configurations.

Canal configuration was classified according to the following Vertucci criteria^13^ (Fig. 1):

1. Type I: A single canal appears from the pulp chamber to the apex.
2. Type II: 2 separate canals leave the pulp chamber but merge into 1 at exit.
3. Type III: 1 canal leaves the pulp chamber, divides into 2 within the root, and then merges to the exit.
4. Type IV: 2 distinctly separate canals are present from the pulp chamber to the apex.
5. Type V: A single canal leaves the pulp chamber but divides into 2.
6. Type VI: 2 separate canals leave the pulp chamber, join at the midpoint, and then divide again into 2 with 2 separate apical foramina.
7. Type VII: 1 canal leaves the pulp chamber, divides and then rejoins within the root, and finally redivides into 2 separate canals with 2 separate apical foramina.
8. Type VIII: 3 separate and distinct canals begin from the pulp chamber and extend to the root apex.

### The standard consistency test (kappa value)

For reliability testing, an endodontist and a radiologist, both highly experienced, evaluated all samples twice within a 6 week interval period. At two separate times, a standard consistency check (kappa value) of the results was performed.

Reliability was considered unqualified when the kappa value was #0.4; reliability was considered moderate when the kappa value was between 0.41 and 0.6; reliability was considered excellent when the kappa value was between 0.61 and 0.8; and reliability was considered fully reliable when the kappa value was between 0.81 and 1.0.^14^

### Statistical analysis

The collected data were inputted to the Statistical Package of Social Sciences software program for Windows (SPSS version 25, Chicago, II, USA) for coding and analysis. The number of roots, the number of canals, and canal system configurations of all mandibular anterior permanent teeth were evaluated. The differences by gender were assessed for the above-mentioned variables. The results were expressed as frequencies and percentages. A Chi-square test was applied to assess differences between males and females in root canal configuration as well as the effect of age on root canal number. The level of significance for all statistical tests was set at p-value < 0.05.

## Results

The outcome of the examiners' accuracy review of the readings was 0.856, suggesting that in this study; the clinical information was fully accurate.

**Figure 2:**
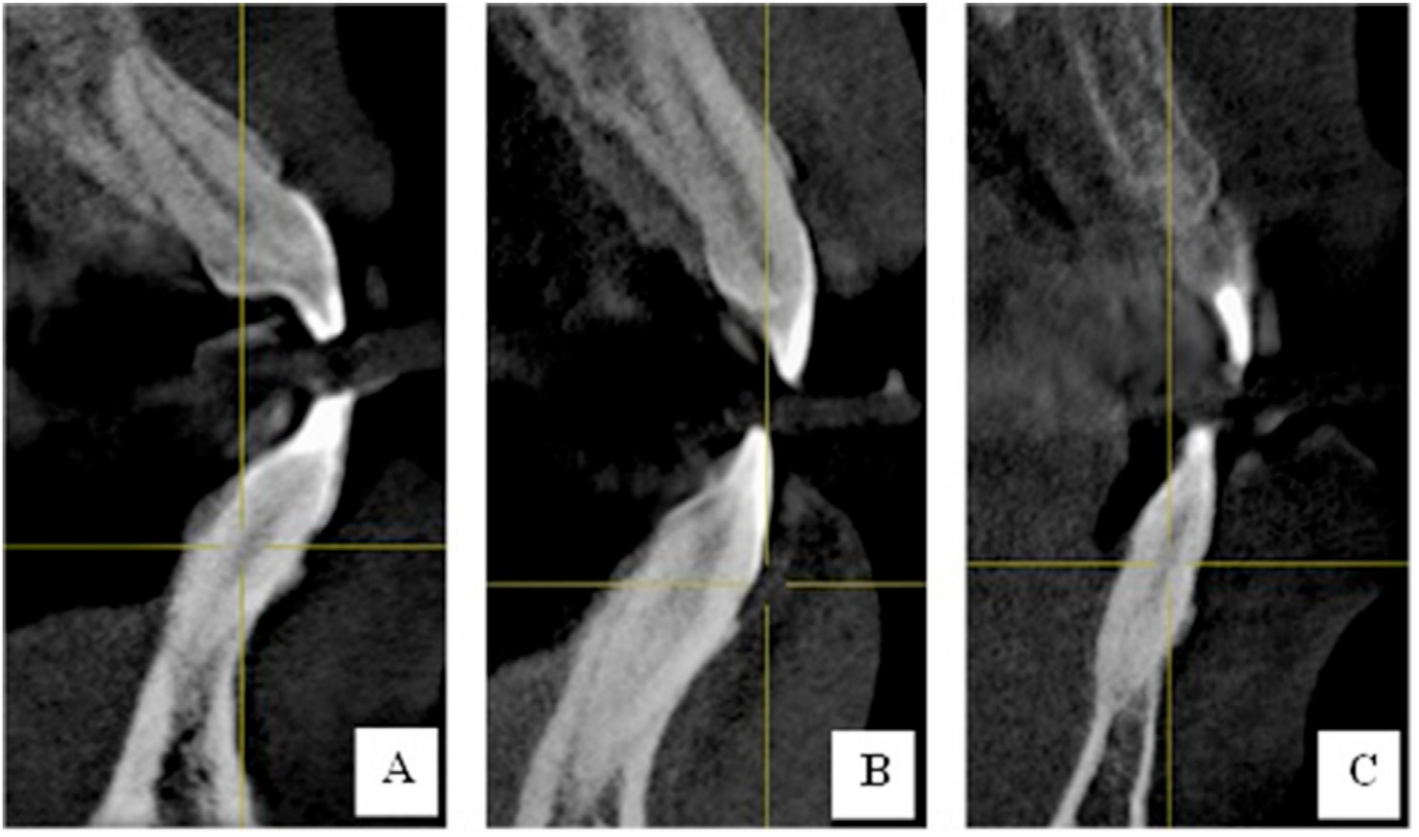
CBCT coronal section of mandibular central incisor (A: Type I; B: Type II; and Type III Vertucci's classification).

**Figure 3:**
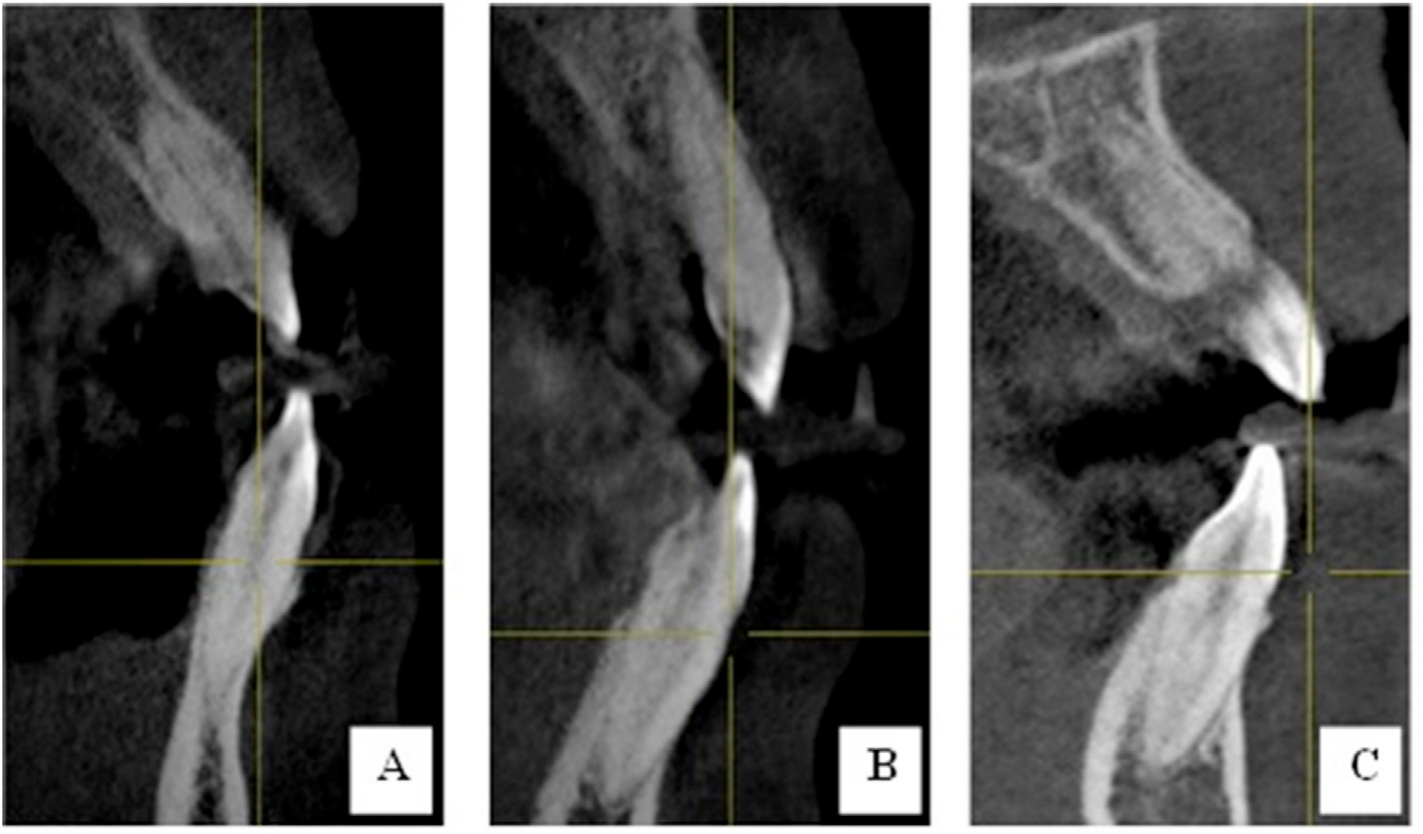
CBCT coronal section of mandibular lateral incisor (A: Type I; B: Type II; and Type III Vertucci's classification).

CBCT scans from 305 patients were evaluated. Among these patients, 122 were males and 183 were females. A total of 1794 mandibular anterior teeth were further investigated to detect the canal configurations using Vertucci's classification. All mandibular central and lateral incisors had a single root (with only Type I, II, and III Vertucci canal configuration (Figure 2, Figure 3). Meanwhile, 2.3% of the mandibular right canines and 2% of the mandibular left canines had two roots (with Type I, II, III, and V Vertucci canal configuration (Figure 2, Figure 3).

**Figure 4:**
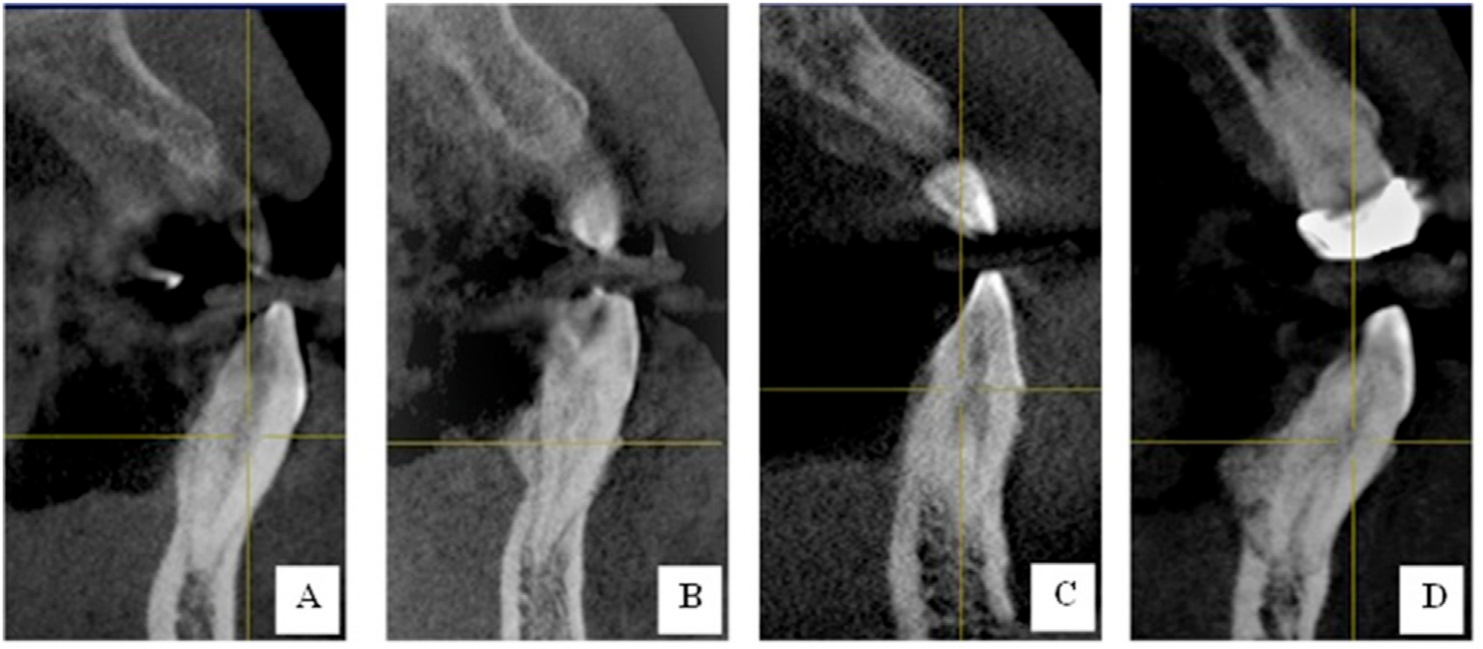
CBCT coronal section of mandibular canine (A: Type I; B: Type II; Type III; and Type V Vertucci's classification).

Additionally, second root canals were detected in 400 cases (22.2%) among the mandibular anterior teeth, Table 1. Lateral incisors presented more commonly with second root canal compared to central incisors and canines on both sides. Canines presented as the anterior teeth with the least prevalence of the two root canals (right 35 (11.7%) and left 33 (11%)), as shown in Table 1. Overall, Type I was the most frequent configuration in all mandibular anterior teeth, followed by Type II, Type III and Type V. Among the examined teeth, 74.1% of mandibular right central incisors and 73.5% of mandibular left central incisors were Vertucci type I. Meanwhile, 73% of mandibular right lateral incisors and 69.5% of mandibular left lateral incisors were Vertucci type I. Moreover, 87.9% of mandibular right canines and 88.9% of mandibular left canines were Vertucci type I. Finally, Vertucci type V presented only in canines (3% in right canine and 2.3% in left canine), as shown in Table 2.

**Table 1:** Number and percentage of root and root canals in mandibular anterior teeth.

Tooth number	Root number		Canal number		Total
	One Root	Two Roots	One Canal	Two Canals	
31	299 (100%)	0	200 (66.88%)	79 (26.4%)	299
32	299 (100%)	0	208 (69.5%)	91 (30.4%)	299
33	293 (97.9%)	6 (2%)	266 (88.9%)	33 (11%)	299
41	298 (100)	0	220 (73.8%)	78 (26.1%)	298
42	300 (100%)	0	216 (72%)	84 (28%)	300
43	292 (97.6%)	7 (2.3%)	264 (88.2%)	35 (11.7%)	299
Total	1781 (99.2)	13 (0.7%)	1394 (77.7%)	400 (22.2%)	1794

Notes: 31, left mandibular central incisor; 41, right mandibular central incisor; 32, left mandibular lateral incisor; 42, right mandibular lateral incisor; 33, left mandibular canine; 43, right mandibular canine.

**Table 2:** Number and percentage of root canal configurations of mandibular anterior teeth according to Vertucci's classification.

Tooth number	Vertucci classification				Total
	Type I	Type II	Type III	Type V	
31	220 (73.5%)	55 (18.3%)	24 (8%)	0	299
32	208 (69.5%)	62 (20.7%)	29 (9.6%)	0	299
33	266 (88.9%)	14 (4.6%)	12 (4%)	7 (2.3%)	299
41	221 (74.1%)	53 (17.7%)	24 (8%)	0	298
42	219 (73%)	56 (18.6%)	25 (8.3%)	0	300
43	263 (87.9%)	15 (5%)	12 (4%)	9 (3%)	299
Total	1397 (77.8%)	255 (14.2%)	126 (7%)	16 (0.8%)	1794

Notes: Type I (A single canal extends from the pulp chamber to the apex); Type II (Two separate canals leave the pulp chamber and join short of the apex to form one canal); Type III (One canal leaves the pulp chamber and divides into two within the root, and then merge to exit as one canal); and Type V (One canal leaves the pulp chamber and divides short of the apex into two separate and distinct canals with separate apical foramina).

Significant differences were identified between males and females concerning root canal configuration for both central incisors (right P = 0.040 and left P = 0.044). In female patients, 141 (79.2%) right mandibular central incisors and 141 (78.7%) left mandibular central incisors showed Type I Vertucci configuration, while 80 (66.6%) right mandibular central incisor and 79 (65.8%) left mandibular central incisors showed Type I Vertucci configuration among male patients, as shown in Table 2. Type II configuration in both right and left mandibular central and lateral incisors was more commonly detected in males than females, but with no statistically significant differences (p > 0.05). Moreover, Type V was more prevalent in males than females in both right and left canines, again with no significant differences (right p = 0.271 and left p = 0.245) (Table 3).

**Table 3:** Number and percentage of Vertucci's root canal configurations of mandibular incisors according to tooth type and sex.

Tooth number			Vertucci classification				Total	Chi-square test
			Type I	Type II	Type III	Type V		
31	sex	Male	79 (65.8%)	29 (24.1%)	12 (10%)	0	120	0.044
		Female	141 (78.7%)	26 (14.5%)	12 (6.7%)	0	179	
		Total	220 (73.5%)	55 (18.3%)	24 (8%)	0	299	
32	sex	Male	75 (62.5%)	32 (26.6%)	13 (10.8)	0	120	0.078
		Female	133 (74.3%)	30 (16.7%)	16 (8.9%)	0	179	
		Total	208 (69.5%)	62 (20.7%)	29 (9.6%)	0	299	
33	sex	Male	107 (89.1%)	7 (5.8%)	2 (1.6%)	4 (3.3%)	120	0.245
		Female	159 (88.8%)	7 (3.9%)	10 (5.5%)	3 (1.6%)	179	
		Total	266 (88.9%)	14 (4.6%)	12 (4%)	7 (2.3%)	299	
41	Sex	Male	80 (66.6%)	29 (24.1%)	11 (9.1%)	0	120	0.040
		Female	141 (79.2%)	24 (13.4%)	13 (7.3%)	0	178	
		Total	221 (74.1%)	53 (17.7%)	24 (8%)		298	
42	sex	Male	79 (65.8%)	29 (24.1%)	12 (10%)	0	120	0.070
		Female	140 (77.7%)	27 (15%)	13 (7.2%)	0	180	
		Total	219 (73%)	56 (18.6%)	25 (8.3%)	0	300	
43	sex	Male	106 (88.3%)	7 (5.8%)	2 (1.6%)	5 (4.1%)	120	0.271
		Female	157 (87.7%)	8 (4.4%)	10 (5.5%)	4 (2.2%)	179	
		Total	263 (87.9%)	15 (5%)	12 (4%)	9 (3%)	299	
Total	sex	Male	526 (73%)	133 (18.4%)	52 (7.2%)	9 (1.2%)	720	0.000
		Female	871 (81%)	122 (11.3%)	74 (6.8%)	7 (0.6%)	1074	
		Total	1397 (77.8%)	255 (14.2%)	126 (7%)	16 (0.8%)	1794	

All mandibular central and lateral incisors had a single root, while 2.3% of the mandibular right canines and 2% of mandibular left canines had two roots. Moreover, second root canals were detected in 400 (22.2%) of the total mandibular anterior teeth (Table 3). Lateral incisors presented more commonly with second root canal compared to central incisors and canines on both sides. Canines presented as the anterior teeth with the least prevalence of the two root canals (right 35 (11.7%) and left 33 (11%)), as shown in Table 3.

The effect of age was examined for the number of canals only. Statistically significant differences were found between patients over 60 years and patients in the other age groups, with a lower frequency of two root canals being detected among patients over 60 years (p ≤ 0.001) (Table 4).

**Table 4:** Number and percentage of root and root canals in mandibular anterior teeth in different age categories.

Age group	Canal number		Total	Chi-square test
	One Canal	Two Canals		
18–29	426 (30.6%)	108 (27%)	534 (29.8%)	P < 0.001
30–39	282 (20.2%)	101 (25.3%)	383 (21.3%)	
40–49	407 (29.2%)	80 (20%)	487 (27.1%)	
50–59	201 (14.4%)	75 (18.8%)	276 (15.4%)	
≥60	78 (5.6%)	36 (9%)	114 (6.4%)	
Total	1394 (100%)	400 (100%)	1794 (100%)	

When comparing the right and left mandibular anterior teeth, central and lateral incisors showed symmetrical and identical root numbers (100%), while canines showed (99.5%) symmetry in root number between the right and left. CBCT images revealed differences (99.87% canines; 99.83% central incisors; and 98.5% lateral incisors) in the numbers of root canals between right and left as shown in Table 5.

**Table 5:** Number and percentage of symmetry of root and root canals in mandibular anterior teeth.

Teeth	Bilateral symmetry cases for root number	Bilateral symmetry cases for root canal number	Total
Central Incisor	597 (100%)	596 (99.83%)	597
Lateral Incisor	599 (100%)	590 (98.5%)	599
Canine	595 (99.5%)	596 (99.87%)	598

## Discussion

In this study, CBCT was used in preference to conventional radiography to investigate the number of roots, root canals, and canal configuration in permanent mandibular anterior teeth as it allows in vivo three-dimensional evaluation of the root canals.^15^ The level of accuracy in the description of the root canal configuration was 0.55 for radiography and 0.89 for CBCT, and the values for sensitivity and specificity were 0.18 and 0.79 and 0.93 and 0.98, respectively.^16^

Although several other classifications have been described in the literature,^17^^,^^18^ in the present study, the Vertucci classification was used to evaluate the root canal configuration as it seems to be the most frequently used classification.^4^^,^^19^^,^^20^ Root canal configurations of mandibular incisors and canines have been extensively studied by CBCT as shown in Table 6. In the present study, only the Vertucci types I, II, III, and IV were seen in mandibular incisors and canines. Type 1 Vertucci configuration was the most prevalent configuration, which was in accordance with previous studies by Altunsoy et al. 19, Basha et al.,^25^ and Verma et al.,^30^ and in contrast to the study conducted by Mashyakhy,^22^ which reported that type III was the most prevalent configuration. Additionally, in the present study, type V canal configuration was the least prevalent type, found only in canines, which is in accordance with the Haghanifar et al.^31^ and Doumani et al.^32^

**Table 6:** Percentages of root canal systems found in mandibular anterior teeth in previous studies assessed by CBCT.

References	Region/race	Number of teeth	Teeth studied	Types									Symmetry
				I	II	III	IV	V	VI	VII	VIII	Multi-rooted	
Aminsobhani et al.^21^	Iran	632	Mandibular central incisor	72.7	11.3	4.7	7.7	3.6	0	0	0	100	–
		614	Mandibular lateral incisor	70.6	7.1	3.7	15.4	3.2	0	0	0	100	–
		608	Mandibular canines	71.8	10.3	2.8	12.8	2.3	0	0	0	4.7	–
Mashyakhy et al.^22^	Saudi Arabia	410	Mandibular central	73.7	0	26.3	0	0	0	0	0	–	Bilateral symmetry in root canal number: 91.2%
		412	Mandibular lateral	69.2	0	29.8	0	1	0	0	0	–	Bilateral symmetry in root canal number: 85.8%
Ying et al.^23^	Beijing, Republic of China	1566	Mandibular central incisor	93.30	0	5.68	0	1.02	0	0	0	100	
		1566	Mandibular lateral incisor	82.57	0	15.52	0	1.85	0	0.06	0	100	
		1542	Mandibular canines	97.02	0	2.20	0	0.71	0	0.06	0	0.7	
Arslan et al.^24^	Turkey	184	Mandibular central incisor	51.9	4.3	41.6	0.5	0	0	0	0	–	
		190	Mandibular lateral incisor	52.9	2.6	42.3	1.6	0	0	0	0	–	
Basha^25^	Egypt	200	Mandibular central incisor	85.5	0	11	0	3.5	0	0	0	–	
		200	Mandibular lateral incisor	89.5	0	8	0	2.5	0	0	0	–	
		200	Mandibular canines	100	0	0	0	0	0	0	0	–	
Baxter et al.^26^	Germany	604	Mandibular central incisor	76.1	22	0	0.6	1.1	0	0	0	–	Type I: 77%; Rest of types: 17.5%
		604	Mandibular lateral incisor	76.6	21.3	0	0.9	0.9	0	0	0	–	77%; Rest of types: 20.5%
Popovic et al.^27^	Serbia	296	Mandibular central incisor	73.0	4.7	21.6	0	0.7	0	0	0	100	–
		294	Mandibular lateral incisor	73.5	5.4	18.4	0.7	2	0	0	0	100	–
		312	Mandibular canines	92.9	0.6	0.6	0	5.8	0	0	0	5.8	–
Valenti-Obino et al.^28^	Italy	487	Mandibular central incisor	55	34.3	9.3	0.6	0.8	0	0	0	100	Bilateral symmetry in root canal morphology: 44.6%
		491	Mandibular lateral incisor	57	35.7	6.9	0	0.4	0	0	0	100	Bilateral symmetry in root canal morphology: 44.8%
Da Silva et al.^29^	Brazil	200	Mandibular central incisor	64.5	0	18	0	14.5	0.5	2.5	0	–	–
		200	Mandibular lateral incisor	60.5	0.5	25.5	0	12	0	1.5	0	–	–
Present study	Iraq	597	Mandibular central incisor	73.8	18	8	0	0	0	0	0	100	Bilateral symmetry in root number: 100%; bilateral symmetry in root canal number: 99.83%
		599	Mandibular lateral incisor	71.2	12.8	6.8	0	0	0	0	0	100	Bilateral symmetry in root number: 100%; bilateral symmetry in root canal number: 98.5%
		598	Mandibular canines	88.4	4.8	4	0	2.6	0	0	0	2.1	Bilateral symmetry in root number; 99.5%; bilateral symmetry in root canal number: 99.87%

Notes: CBCT; Cone-beam computed tomography.

The presence of two roots in a mandibular incisor is a rare finding.^20^^,^^33^ In this study, all mandibular incisors on both sides had one root. Meanwhile, two-rooted mandibular canines were found in only around 2% of cases, which is in agreement with previous findings that reported a prevalence range of 0.3–6.2%.^21^^,^^34^ The percentage of number of roots in previous studies comparing to present study are shown in Table 6.

Compared with the prevalence of two roots, previous studies have found greater variations in the prevalence rates for two root canals in incisors (8–33%) and canines (9–30%).^20^^,^^22^^,^^26^^,^^27^^,^^30^^,^^35^^,^^36^ These variations may be due to ethnic differences and sample size. The present study found a rate of 26.1%–26.4% for two root canals in central incisors, whereas the rate for lateral incisors was 28%–30.4%, and the finding for canines was 11–11.7%. Compared to other studies in Iraqi population, the findings of the current study for the prevalence rates of two root canals in incisors and canines are very similar to those of Goran and Rofoo^37^ (33.1% in central incisors, 33.1% in lateral incisor, and 9.2% in canines) and Dizayee and Selman^38^ (20% for incisors).

Anatomical symmetry in tooth properties is clinically important when treating contralateral teeth in the same patient.^39^ More symmetry in terms of the number of roots in the mandibular anterior teeth was seen in incisors compared to canines. However, the overall symmetry in the root canal numbers was more prevalent in canines and central incisors (99.8%) than in lateral incisors (98.5%), compared to 90–95% in other studies.^28^^,^^34^^,^^36^ Clinically, this implies that when two-canalled mandibular anterior tooth has been detected and treated, the contralateral teeth will, with high probability, also have two root canals with an identical or at least similar configuration.

Among the demographic variables, age displayed an inverse relationship with the frequency of the two root canals, probably due to the deposition of secondary dentin in the root canal, which may ultimately result in obliteration of the root canal space and impede the radiographic appearance of these structures.^40^^,^^41^

Only three studies^34^^,^^36^^,^^42^ investigated the relationship between age and the number of the root canals in the mandibular anterior teeth. In one of these studies, no significant difference was found between the frequencies of two root canals in the teeth of patients in various age groups.^36^ In the present study, significant differences were found between patients over 60 years and patients in the other age groups.

According to gender, type I Vertucci configurations in permanent mandibular central incisors were significantly more common in females than males. However, in both right and left mandibular central and lateral incisors, type II configuration was more commonly detected in males than females, but with no statistically significant difference, which is in accordance with the Liu et al.^43^ and Chellammal^44^ studies and in contrast to the study conducted by Zhengyan et al.^14^ The differences in the results may be due to variations in sample size and ethnic origin. Under the limitations of this study, CBCT imaging has been demonstrated as an excellent method for the detection of different canal configurations of mandibular anterior teeth and the findings obtained emphasize the importance of knowledge of variations in root canal morphology, since excluding the possibility of morphological variation can lead to failure of endodontic therapy.

In conclusions, around 22% of mandibular anterior teeth have two root canals, but only mandibular canines present with two roots, also type I Vertucci configuration was the most prevalent and type V was found only in mandibular canines.

## Authorship

Ranjdar Talabani: Conception and design of study, acquisition of data_,_ Analysis and/or interpretation of data, Drafting the manuscript, revising the manuscript critically for important intellectual content.

## Declaration of competing interest

The authors have no conflict of interest to declare.
